# Loss of BAX by miR-365 Promotes Cutaneous Squamous Cell Carcinoma Progression by Suppressing Apoptosis

**DOI:** 10.3390/ijms18061157

**Published:** 2017-05-30

**Authors:** Liang Zhou, Ruirui Gao, Yinghui Wang, Meijuan Zhou, Zhenhua Ding

**Affiliations:** Department of Radiation Medicine, Guangdong Provincial Key Laboratory of Tropical Disease Research, School of Public Health, Southern Medical University, Guangzhou 510515, China; zhzliang@smu.edu.cn (L.Z.); rui10520@163.com (R.G.); wyh045@i.smu.edu.cn (Y.W.); lkzmj@smu.edu.cn (M.Z.)

**Keywords:** miR-365, cutaneous squamous cell carcinoma, BAX, apoptosis

## Abstract

Pro-apoptotic BCL2 associated X (BAX) is traditionally thought to be regulated by anti-apoptotic BCL-2 family members, like BCL2-like 1 (BCL-XL), at the protein level. However, the posttranscriptional regulation of BAX is under explored. In this study, we identified BAX as the novel downstream target of miR-365, which is supported by gain- and loss-of-function studies of onco-miR-365. Loss of BAX by either RNA interference or highly-expressed miR-365 in cells of cutaneous squamous cell carcinoma (CSCC) enhanced the tumor resistance against apoptosis, while repressing cell proliferation, migration, and invasiveness. In vivo experiment confirmed that BAX knockdown promotes the growth of CSCC xenografts. Collectively, our results find a miR-365-BAX axis for alleviating the pro-apoptotic effects of BAX, which promotes CSCC development and may facilitate the generation of novel therapeutic regimens to the clinical treatment of CSCC.

## 1. Introduction

Cutaneous squamous cell carcinoma (CSCC) is the second-most common cancer with an annual incidence of more than one million cases worldwide [1]. Chronic sun exposure, which means a higher cumulative dose exposure of ultraviolet (UV) rays, can damage the DNA of normal keratinocytes in the epidermis, leading to the development of skin cancer, including CSCC [1,2]. However, the underlying molecular mechanism(s) responsible for this transition remain obscure.

MicroRNA is a class of highly-conserved single chain noncoding RNA, 19~25 nt in length. In the past decade, the expression profiles, functions, and mechanism of microRNA in cancer have been well explored, and evidence has supported the proposition that microRNAs are widely involved in the key processes of carcinogenesis and the further development of cancers [3,4]. However, studies about the roles of miRNAs in CSCC are not vigorous. Differentially-expressed microRNAs, including the upregulated miRNAs (miR-135b, miR-424, miR-766) and downregulated microRNAs (miR-30a*, miR-378, miR-145, miR-140-3p, miR-30a, and miR-26a), have been identified by microarray analysis in tumor tissues from patients harboring CSCC compared with normal skin tissues [5]. miR-125b can target the matrix metalloproteinase MMP13 to inhibits the proliferation, migration, and invasion of CSCC [6]. miR-31 can promote the growth of CSCC by targeting the tumor suppressor gene LATS2 [7]. These studies indicate that microRNAs are pivotal players in CSCC development, while the mechanistic aspects are under-explored.

Apoptosis, a programmed cellular process constituting one of the most researched topics among cell biologists, occurs in physiological and pathological conditions [8]. Originally, apoptosis was defined as one of the defensive weapons among the human arsenal for countering cancer invasion [9]. The BCL-2 family, whose members are either pro-apoptotic (BAX, BCL2 antagonist/killer 1 [BAK1], BH3 interacting domain death agonist [BID], and BCL2 like 11 [BIM]) or anti-apoptotic (B cell leukemia/lymphoma 2 [BCL-2], BCL-XL, and BCL2 like 2 [BCL-W]), governs the mitochondrial-mediated programmed death signaling by regulating cytochrome C release. One of the known tumor suppressors, p53, can trigger apoptosis by upregulating of pro-apoptotic BAX to release cytochrome C [9]. However, cancers develop many mechanisms to evade apoptosis, which becomes one of the hallmarks of cancer [8]. As one of mitochondrial pro-apoptotic factors, BAX is under intensive investigations and has been shown to have special importance in cancer therapy by regulating its expression and activities [10].

Our previous studies have shown that onco-miR-365 is one of the abbrently-regulated miRNAs induced by UV treatment which is highly expressed in both CSCC cell lines and clinical samples [11,12]. Evidence also supports the role of miR-365 in the malignant transformation of keratinocytes, promoting the development of CSCC, and inhibition of miR-365 by antagomiR oligo treatment that can repress CSCC tumor formation in vivo [11,12]. The identified downstream targets of miR-365 include cell division cycle 25A (CDC25A), cyclin D1 (CCND1), transcription termination factor 1 (TTF1), interleukin 6 (IL-6) and nuclear factor I B (NFIB) in various types of cancers [13,14,15,16,17,18,19]. In view of the regulatory functions of microRNA are performed through targeting multiple downstream genes, it is necessary to investigate the important downstream targets to unveil all of the critical roles performed by this microRNA, which may also improve our understanding of mechanistic regulation of microRNAs in CSCC. Importantly, the above studies indicated that miR-365 can inhibit apoptosis [11,13]. Yet, how miR-365 regulating apoptosis is unclear.

In this study, we begin an investigation into downstream apoptotic regulators of miR-365 to address the above question. BAX, a pro-apoptosis regulator, was originally predicted as the direct downstream target of miR-365 by bioinformatic analysis. Evidence from gain- or loss-of-function experiments supported the proposition that BAX is the direct downstream target of miR-365 and regulated at a posttranscriptional level. Loss of BAX by RNA interference in CSCC enhanced the tumor proliferation, migration, invasiveness, and resistance against apoptosis. In vivo animal experiments confirmed that depletion of BAX promotes tumor growth. Collectively, the miR-365-BAX axis identified here not only deepens our understanding of the pivotal roles of microRNAs in CSCC progression, but also may provide potential targets for developing novel anti-cancer drugs and contribute to the establishment of new therapeutic regimes.

## 2. Results

### 2.1. BAX is Predicted to Be the Direct Target of miR-365 and Downregulated in CSCC Cells and Tumors

As our previous study has confirmed the highly expression of miR-365 in CSCC cell lines and tumors [7], it is critical to identify the downstream targets for dissecting the molecular events regulated by miR-365. Thus, we screened and found that BAX was consistently predicted to be one of the downstream targets of miR-365 by web-based software, including TargetScan [13], miRanda [14], and miRDB [15]. Importantly, as a known apoptosis regulator, the role of BAX in CSCC tumorigenesis is underexplored.

**Figure 1:**
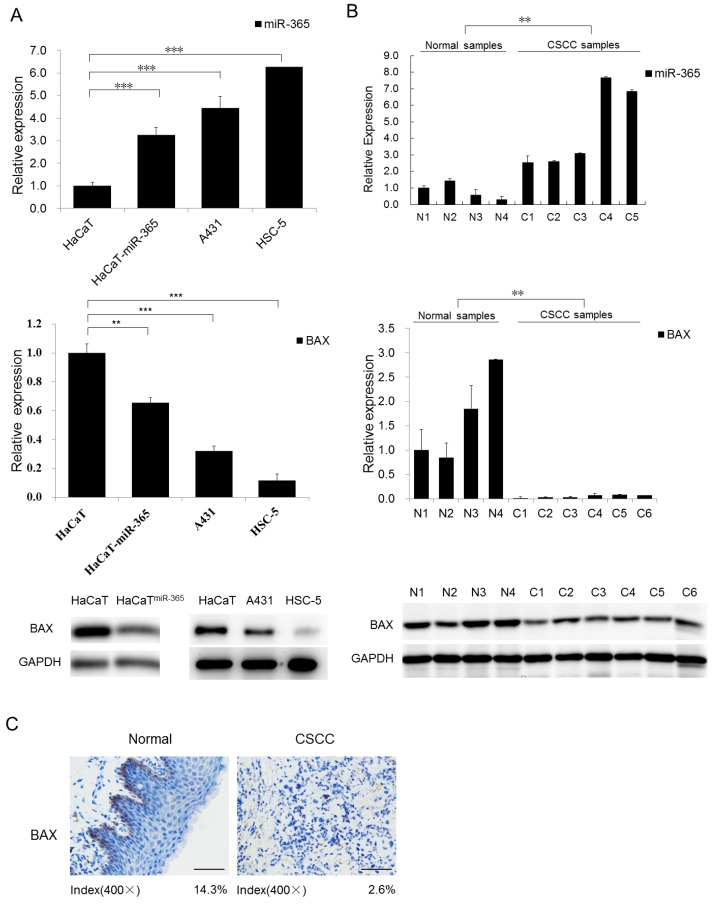
BAX is decreased in cutaneous squamous cell carcinoma (CSCC) primary tumors and cell lines. (**A**) BAX and miR-365 expression was detected in the CSCC cell lines A431, HSC-5, and HaCaT^miR-365^ compared with HaCaT keratinocytes in both mRNA and protein levels. The qPCR results were evaluated by normalizing to U6 snRNA (for miR-365) or GAPDH (for BAX). In Western blot, GAPDH was detected for using as loading control; (**B**) BAX and miR-365 expression were detected in normal tissues (N1–N4) and CSCC primary tumors (C1–C6) in both mRNA and protein levels; (**C**) IHC detection of BAX on paraffin sections of CSCC tumors and normal skin specimens. Positive signals were shown in brown staining (magnification, 400×), scale bars, 50 µm. The percentage of positive staining was marked for each group. ** *p* < 0.01, *** *p* < 0.001.

To probe into the possible involvement of BAX in CSCC development and progression, we began by examining the expression levels of BAX in CSCC cell lines, A431, HSC-1, and malignant transformed cell strain HaCaT^miR-365^, which were compared with benign HaCaT keratinocytes. We checked the miR-365 expression in the above cell lines which is inversely correlated with BAX expression in those cell lines at the mRNA level (Figure 1A). Western blot results showed that the expression of BAX was significantly downregulated in CSCC cell lines and HaCaT^miR-365^ cell strain when compared with the HaCaT keratinocytes (Figure 1B). We also detected BAX expression in clinical samples and found that BAX is significantly downregulated in clinical patient CSCC samples compared with normal skin tissues (Figure 1C), showing an inverse correlation with miR-365 expression in CSCC as shown in our previous studies using the same CSCC samples [11]. Collectively, the above results suggest that BAX is significantly less expressed in both primary tumors and CSCC cells, which is inversely correlated with highly-expressed miR-365.

### 2.2. miR-365 Directly Targets BAX through Binding to Its 3′-UTR Region

**Figure 2:**
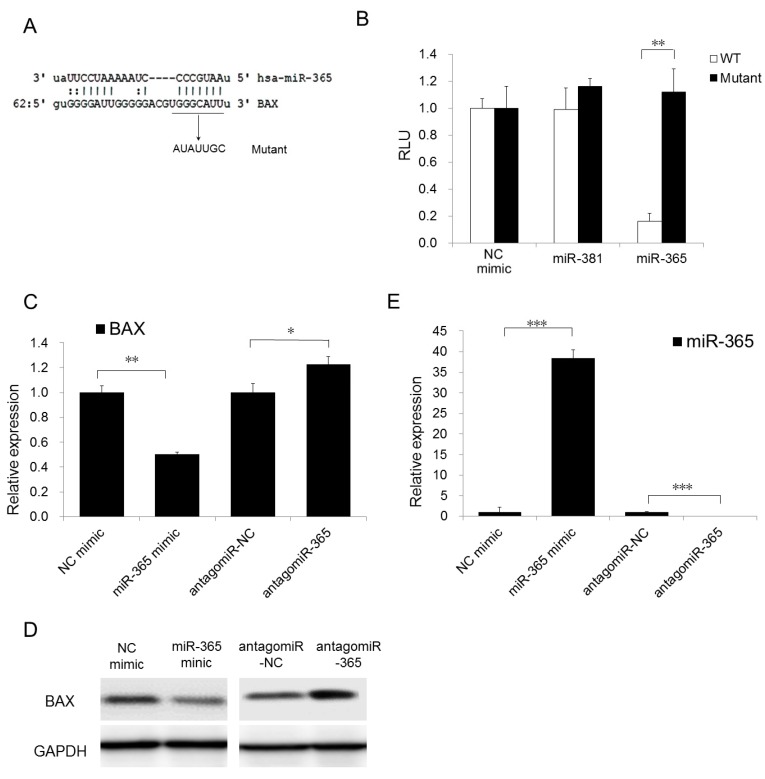
miR-365 and its putative binding sequence in 3′-UTR of BAX. (**A**) A schematic illustration of base pairing between miR-365 with the 3′ UTR of BAX. The mutated sequences were underlined; (**B**) WT or mutant reporter constructs were then transfected into A431 cells with NC, miR-365 mimics; and miR-381 mimics. Dual luciferase assay was performed 48 h post-transfection and normalized to Renilla luciferase activities. Data represent the average of three independent experiments ± s.d.; (**C**,**D**) BAX mRNA and protein expression was measured in NC mimic, miR-365 mimic, antagomiR NC, or antagomiR-365 transfected A431 cells by qRT-PCR and normalized with GAPDH. Expression folds are shown with respect to NC mimic or antagomiR NC cells where normalized copy numbers were set to 1. In Western blot, GAPDH was detected for using as loading control; (**E**) miR-365 expression was verified by qPCR. The qPCR results were evaluated by normalizing to U6 snRNA. * *p* < 0.05, ** *p* < 0.01, *** *p* < 0.001.

To investigate if the downregulation of BAX is owing to miR-365 direct targeting, an evolutionarily-conserved miR-365 binding site was identified within the BAX 3′-UTR (Figure 2A). The fragments of the wildtype BAX 3′-UTR regions containing the miR-365 target site was cloned into a reporter plasmid and placed downstream of the firefly luciferase reporter gene. This reporter plasmid was cotransfected together with miR-365 and this caused repression of the luciferase activity (Figure 2B). Such a targeting effect was specific to miR-365 binding because the reporter activity was shown to not be affected when transfections were repeated with an irrelevant miRNA, miR-381, or with the reporter containing a mutant miR-365 binding site in the BAX 3′-UTR (Figure 2B). Together, miR-365 specifically targets the *BAX* gene.

### 2.3. BAX Expression Is Inversely Correlated with miR-365 Expression

To check if BAX expression is regulated by miR-365 in CSCC cells, we assumed that the binding of miR-365 to BAX 3′-UTR may lead to the repression of BAX expression. As we predicted, overexpression of miR-365 led to downregulation of BAX expression, while the knocking down of miR-365 by antagomiR-365 could upregulate the expression of BAX in both mRNA and protein levels (Figure 2C,D), while the miR-365 expression was verified at the same time (Figure 2E). Together, the above results indicated that BAX is one of the direct downstream targets of miR-365 and depletion of miR-365 can abolish the repression and cause the upregulation of BAX.

### 2.4. Loss of BAX Promotes CSCC Cell Proliferation, Migration, and Invasiveness, but Represses Apoptosis

**Figure 3:**
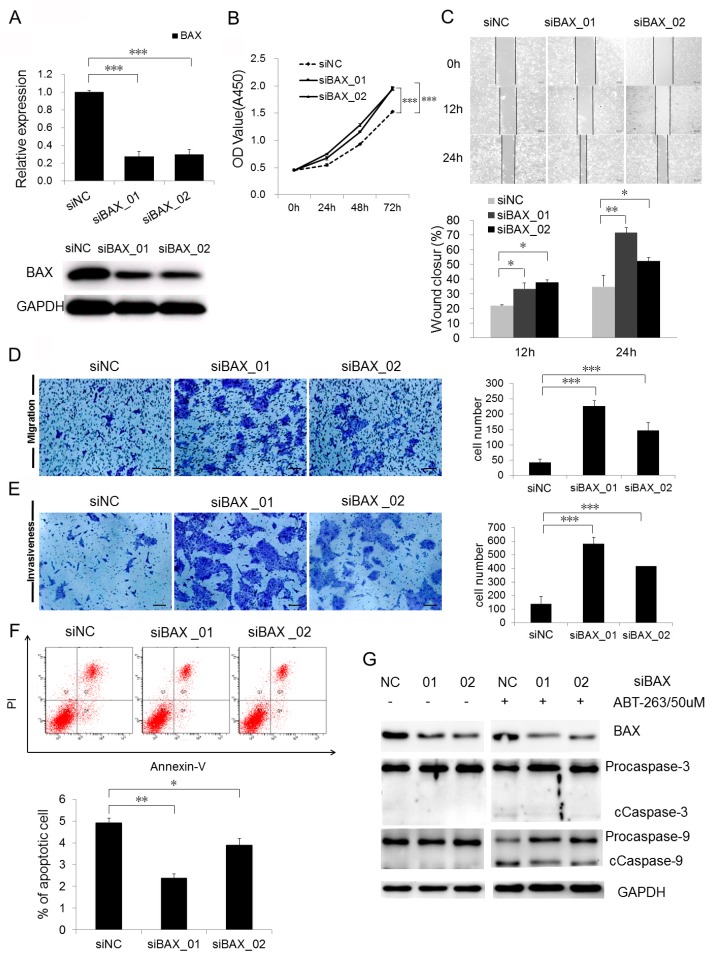
Loss of BAX promotes CSCC cell proliferation, migration, and invasiveness, but represses apoptosis. (**A**) BAX protein expression was detected after depletion of BAX by siRNAs in A431 cells. Measurement of cell proliferation by CCK-8 assay; (**B**) wound healing assay (magnification, 100×, scale bar, 20 µm); (**C**) transwell migration assay (magnification, 200×, scale bar, 30 µm); (**D**) matrigel invasiveness measurement (magnification, 200×, scale bars, 30 µm); (**E**) apoptosis assay; (**F**) by Annexin V/PI double staining, and Western blotting; (**G**) were performed in A431 treated with siRNA targeting BAX. * *p* < 0.05, ** *p* < 0.01, *** *p* < 0.001.

The downregulation of BAX by miR-365 overexpression implicated that BAX may have an anti-carcinogenic role in CSCC tumorigenesis. To test this notion, depletion of BAX was achieved by transfecting siRNA oligos against BAX into A431 cells, a cell line derived from an epidermoid (squamous cell) carcinoma [20]. A significant loss of BAX in both mRNA and protein levels was achieved by siBAX-treated cells with respect to siNC transfection (Figure 3A). Cell proliferation was then assessed by CCK-8 assay and showed a significant increase of proliferation capacity after siBAX-treatment with respect to siNC treatment (Figure 3B). Wound-healing assay revealed (Figure 3C) that BAX knockdown significantly enhanced A431 cell motility at both 12 and 24 h, resulting in much faster wound closure of the monolayer. This is also supported by transwell migration assay, in which the cells penetrating the pores of the membrane were significantly greater in number than those in siNC-treated cells (Figure 3D). The invasive ability was analyzed by matrigel invasiveness measurement and the results showed that knockdown of BAX significantly promoted the invasive ability of CSCC cells (Figure 3E). Generally, oncogenic genes can repress apoptosis to promote cancer cell growth [21]. Annexin V/PI double staining revealed that knockdown of BAX induced an increase in the living cell population (Annexin V−/PI−) and an accompanying decrease in the early apoptotic population (Annexin V+/PI−; Figure 3F). It is well known that BAX is upstream of caspase cleavage-induced apoptosis. To strengthen the above findings, we knocked down BAX in A431 cells to check the impacts of BAX on apoptosis. However, it was hard to detect the generation of cleaved caspase-3 and caspase-9 in A431 cells while procaspase-3 and procaspase-9 are highly expressed, which cannot be used to observe BAX function during apoptosis (Figure 3G). In contrast, treatment of A431 cells with ABT-263 (Navitoclax), a pro-apoptosis reagent by inhibiting BCL-2, BCL-XL, and BCL-W, induced the cleavage of procaspase-3 and procaspase-9. Knockdown of BAX could significantly alleviate the generation of cleaved caspase-3 and caspase-9 to increase the amount of procaspase-3 and procaspase-9 (Figure 3G), relatively, which is consistent with the results of the Annexin-V/PI assay. Together, the above data demonstrated that loss of BAX promote cancer growth, migration and invasiveness in CSCC cells by inhibiting BAX-induced apoptosis.

### 2.5. Knockdown of BAX Expression Promotes Tumor Growth In Vivo

**Figure 4:**
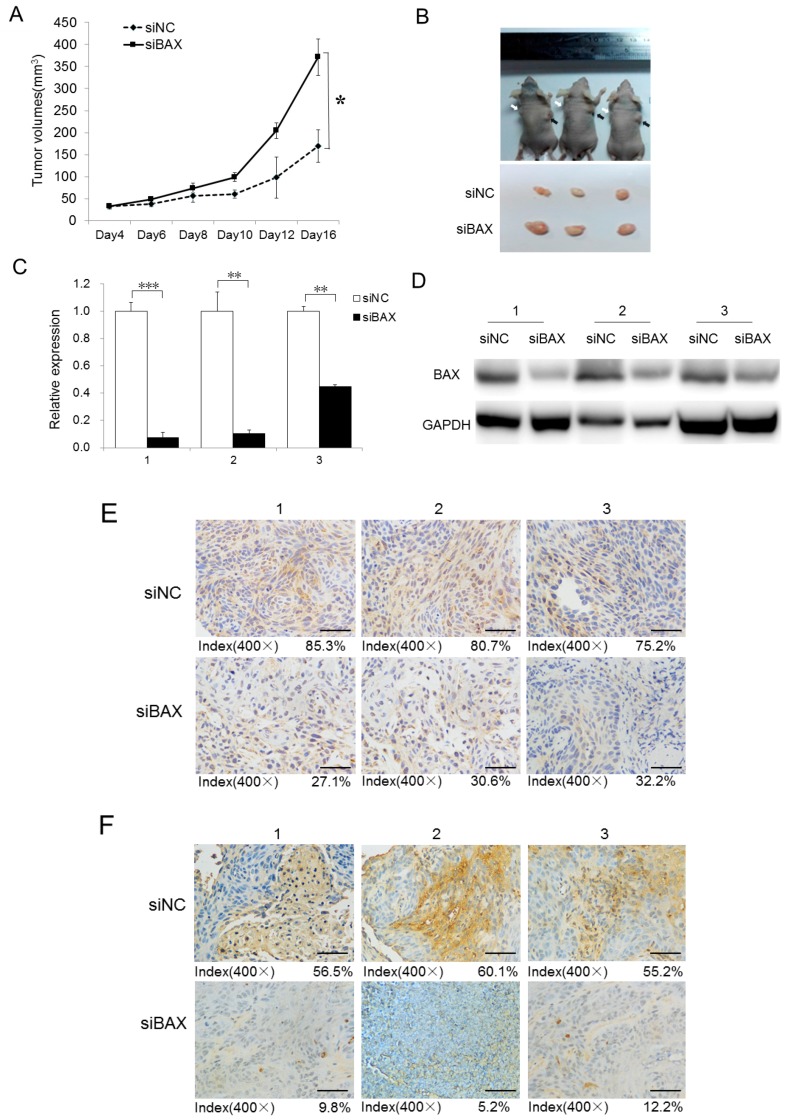
BAX knockdown by siRNA mimics promotes tumor growth in vivo. siNC and siBAX oligos were injected into A431 cell xenografts every three days. (**A**) Loss of BAX promotes subcutaneous tumor growth in a mouse xenograft model. Tumor volumes (mm^3^) were plotted according to days. Tumor volume statistical data represent the average of three independent experiments ± s.d., respectively; (**B**) the mice were sacrificed at the end of the experiment and images taken along with the dissected tumors from three representative mice are shown. White arrows indicate the siNC-treated xenografts, while black arrows indicate siBAX-treated xenografts; (**C**) the expression of BAX was measured in the dissected tumors by qRT-PCR. qRT-PCR statistical data represent the average of three independent experiments ± s.d. Expression folds are shown with respect to NC where normalized copy numbers were set to 1; (**D**) the protein expression of BAX was detected in xenografts after siBAX treatment by Western blot; (**E**) histopathology analysis (IHC staining) of BAX on tumor sections. The quantification was done by counting positively-stained cells from 20 randomly-chosen fields from a total of five sections per tumor. Magnification, 400×, Scale bars, 50 µm; (**F**) TUNEL assay of apoptosis on tumor sections after BAX depletion. The quantification was done by counting positively-stained signals from 20 randomly-chosen fields from a total of five sections per tumor. Magnification, 400×, Scale bars, 50 µm. ** *p* < 0.01, *** *p* < 0.001.

To evaluate the anti-carcinogenic effect of BAX in vivo, the xenograft tumor model was established in immunocompromised mice. When tumor volumes reached approximately 100 mm^3^, 3 nmol control (siNC) or siBAX oligos were directly injected into tumors and the injections were repeated every two days for 16 days. The difference of tumor sizes was not apparent at the very beginning between the two groups. From the tenth day, BAX depletion markedly enhanced tumor growth when compared with the siNC control group. Tumor size and mass were evidently larger at the end of the evaluation (Figure 4A,B) while the loss of BAX expression in the siBAX group was also confirmed by qPCR, Western blot, and IHC staining (Figure 4C–E). The following TUNEL assay for detecting the apoptosis in xenograft tumors indicated that the apoptotic rate was significantly decreased in response to BAX depletion.

## 3. Discussion

In this study, BAX was identified as a novel downstream target of miR-365. Significantly-downregulated expression of BAX is a common feature in CSCC primary tumors and cell lines which show aberrantly-high expression of miR-365. Reporter luciferase assay and gain- and loss-of-function studies of miR-365 confirmed BAX is the downstream target of miR-365. Depletion of BAX by RNA interference imitates the downregulation of BAX by miR-365 upregulation, alleviates apoptotic potential and thus contributes to the development of CSCC.

Apoptosis is evolutionarily conserved in eukaryotes and essential for development, tissue homeostasis, and the clearance of pathogens [22]. Phenotypically, apoptosis is an ordered cascade of enzymatic events, including DNA fragmentation, nuclear condensation, cell shrinkage, blebbing, and phosphatidylserine externalization [22,23]. Apoptotic processes follow either intrinsic or extrinsic pathways [22]. In contrast to the extrinsic pathway, the intrinsic pathway is activated by intracellular stress or damage signaling including growth factor deprivation, DNA damage or release of the endoplasmic reticulum (ER) Ca^2+^, which results in mitochondrial lysis releasing pro-apoptotic proteins, one of which is cytochrome c, which induces the formation of a death-signaling complex (apoptosome) cleaving caspase-9 and inducing the caspase cascade. BCL-2 family members are functionally divided into pro- and anti-apoptotic proteins on the basis of the presence/absence of BCL-2 homology (BH) domains. They interact with each other at the mitochondria and ER membranes, and finely control the mitochondrial permeabilization.

The pro-apoptotic BCL-2 members, including BAX and BAK, also called apoptotic effectors which contain BH 1-3 domains and act by forming homo-oligomeric pores in the mitochondrial outer membrane leading to the disruption and release of pro-apoptotic proteins for promoting the release of ER Ca^2+^ and pushing the apoptotic process forward [24]. BAX is a central death effector and is required at the decisional stage of apoptosis. Loss of BAX impairs apoptosis and inhibits MYC-induced lymphomagenesis [25]. Mutation of the *BAX* gene is widely observed in genetically unstable cancers of the colorectum, stomach, and endometrium [26]. Previous studies indicated that overexpression of BAX induces apoptosis with loss of mitochondrial potential, an early release of cytochrome c preceding many apoptosis-associated morphological alterations as well as caspase activation and subsequent substrate proteolysis [27]. Small BAX agonists can induce conformational variations by blocking phosphorylation sites to facilitate BAX insertion into mitochondrial membranes and form BAX oligomers, which finally leads to the release of cytochrome c and apoptosis in several types of cancers [28]. The pro-apoptotic activity of BAX is generally regulated by other BCL-2 family members, like BCL-2, BCL-XL, etc. Except for the heterodimer model of BCL-XL, which interacts with BAX, BCL-XL is suggested to retrotranslocate BAX from the mitochondria into the cytosol for inhibiting apoptosis [21].

Recent reports have suggested that BAX is under posttranscriptional regulation. miR-886-5p inhibits apoptosis by targeting BAX expression in human cervical carcinoma cells [29]. BAX is also the downstream substrate of miR-128. Downregulation of miR-128 sensitizes breast cancer cell MDA-MB-231 to chemotherapy [30]. Strikingly, miR-128 induces apoptosis in HEK293T cells by also targeting BAX [31]. However, whether BAX is under post-transcription regulation in CSCC is rarely explored. In this study, BAX is identified as the direct downstream target of miR-365 in CSCC through bioinformatic analysis and experimental validation. Loss of BAX by highly-expressed miR-365 enhanced the CSCC tumor resistance against tumor-suppressive apoptosis. Our results, therefore, establish a novel linkage between highly-expressed miR-365 and pro-apoptotic BAX, the miR-365-BAX axis, which contributes to CSCC development.

## 4. Materials and Methods

### 4.1. Ethics Statement

This study was approved by the Institutional Animal Care and Use Committee (IACUC) of Southern Medical University (Approval code L2016103, 13 September 2016). They are in accordance with the guidelines of the Asian Federation of Laboratory Animal Science Associations (AFLAS) and the National Regulations for the Administration of Affairs Concerning Experimental Animals (8 January 2011).

### 4.2. Cell Culture and Tumor Samples

The CSCC lines A431, HSC-1 (HonSun Biological Co., Ltd., Shanghai, China), the human benign epidermal keratinocyte cell line HaCaT (China Center for Type Culture Collection, Wuhan, China), and the HaCaT^miR-365^ cell strain stably overexpressing miR-365 constructed in our previous study [11] were cultured using DMEM (Dulbecco’s modified Eagle medium) with 10% fetal bovine serum and maintain at 37 °C with 5% CO_2_ in a humidified environment. CSCC primary tumor samples were obtained from patients diagnosed with CSCC from 2009 to 2016 in the departments of dermatology, pathology, and oncology at Nanfang Hospital (Guangzhou, China) and Zhujiang Hospital (Guangzhou, China), affiliated to Southern Medical University (Guangzhou, China).

### 4.3. DNA Constructs

Validation of miRNA targets was performed by cloning the partial BAX 3′-untranslated region (UTR) containing the sequence recognized by the miR-365 core seed region. The oligos carrying restriction sites of SpeI and SacI for cloning were as follows: top, 5′-ctagtggaggggtggggattgggggacgtgggcatttttcttacttttgtaatgagct-3′; bottom, 5′-cattacaaaagtaagaaaaatgcccacgtcccccaatccccacccctcca-3. The oligos for mutating BAX 3′-UTR at the miR-381-3p binding sites were as follows: top, 5′-ctagtggaggggtggggattgggggacgtatattgctttcttacttttgtaatgagct-3′; bottom, 5′-CATCATAGAAATAGAGTGTTTAAGCTTAAGTGACATAAAGTATATCA-3′. The paired oligos were annealed and cloned into the restriction sites of SpeI and SacI of the pMIR-report vector (Promega, Madison, WI, USA).

### 4.4. Isolation of RNA and Quantitative Real-Time PCR (qRT-PCR)

Total RNAs from cells were extracted using TRIzol reagent (Life Technologies, Taipei, Taiwan) according to the manufacturer’s instructions. For mRNA quantification, Reverse transcription (RT) of mRNA samples were performed with a M-MLV 1st Strand Kit (Invitrogen, Carlsbad, CA, USA), Oligo(dT)20 primers (Invitrogen). The qRT-PCR of mRNAs were performed with the SYBR Select Master Mix (Invitrogen) on a LightCycler 96 Detection System (Roche, Basel, Switzerland) using GAPDH for normalization. The following primer sequences were used to amplify the indicated genes: *BAX* (forward [F]: 5′-aagaagctgagcgagtgtctcaa-3′; reverse [R]: 5′-cccatgatggttctgatcagttc-3′), and glyceraldehyde 3-phosphate dehydrogenase (*GAPDH*) (F: 5′-ttgccatcaatgaccccttca-3′; R: 5′-cgccccacttgattttgga-3′). For miRNA quantification, TaqMan MicroRNA Reverse Transcription Kit for miR-365 and U6 (Ambion) was used for reverse transcription. miR-365 and U6 was detected using corresponding TaqMan MicroRNA Assays. Cycling parameters were 95 °C for 10 min, followed by 40 cycles of 95 °C for 15 s and an annealing/extension step at 60 °C for 40 s. Gene expression ΔΔ*C*t values for the mRNAs and miR-365 from each sample were calculated by normalizing the values with the internal controls (*GADPH* and *U6* snRNA, respectively). Fold change was calculated by the equation 2^−ΔΔ*C*t^. All experiments were performed in triplicate.

### 4.5. Immunoblotting and Immunohistochemistry (IHC) Assays

Total protein extracts were generated and individual proteins was detected by Western blot as previously reported [32,33]. The primary antibodies used in this study are list below: BAX (Cell Signaling, Danvers, MA, USA; 1:3000), CASPASE-3 (Santa Cruz Biotechnology, Santa Cruz, CA, USA; 1:3000), CASPASE-9 (Cell Signaling; 1:3000) and GAPDH (Santa Cruz Biotechnology; 1:3000). The following secondary antibodies were also used: anti-mouse IgG-horseradish peroxidase (HRP) and anti-rabbit IgG-HRP (Santa Cruz Biotechnology; 1:5000). The expression of individual proteins was visualized using Luminata Forte Western HRP substrate (Millipore, Billerica, MA, USA).

Xenograft tumor sections or formalin-fixed paraffin-embedded CSCC sections were detected using BAX antibody (Cell Signaling; 1:100). The rersults of IHC sections were imaged using a ZEISS Vert.A1 microscope (Carl Zeiss Jena, Oberkochen, Germany), and 20 representative images for each group were collected for statistical analysis.

### 4.6. Luciferase Reporter Assay

For dual luciferase reporter assays, HEK293T cells were co-transfected with pMIR-report constructs, the Renilla luciferase reporter vector (Promega, Fitchburg, WI, USA), and either miR-365, control mimic, or inhibitors (Guangzhou Ribobio, Guangzhou, China), using Lipofectamine 2000 reagent (Invitrogen, Carlsbad, CA, USA), according to the manufacturer’s instructions. Luciferase activity was measured at 48 h after transfection using the Dual-Luciferase Reporter Assay System (Promega), according to the manufacturer’s protocol. Correction for differences in transfection efficiency was performed by normalizing firefly luciferase activity to total Renilla luciferase. Control miRNA mimic/inhibitor and miR-365 mimic/inhibitor were added at 150 nM concentrations.

### 4.7. Apoptosis Assay

A431 cells were seeded on a 60-mm dish and transfected with siBAX_01, siBAX_02, or siNC oligos and cultured for 48 h. A TransDetect Annexin V-FITC/PI cell apoptosis detection kit (TransGen Biotech, Beijing, China) was applied according to the manufacturer’s protocols. Cell apoptosis was detected and quantified by Guava easyCyte Flow Cytometry System (Millipore, Billerica, MA, USA).

### 4.8. Wound Healing Assay

A431 cells were seeded in six-well cell culture plates and transfected with siBAX_01, siBAX_02, or siNC oligos. A yellow pipette tip was used to make a straight scratch to stimulate a wound. The distances travelled (mobility) were measured at 0, 12, and 24 h after scratching.

### 4.9. Cell Proliferation Assay

A431 cells (4000 per well) cultivated on 96-well plates were transfected with siRNAs, and cell proliferation was detected after 0, 24, 48, and 72 h using a cell counting kit (TransGen Biotech) at 450 nm as described in the manual.

### 4.10. Cell Migration Assay

To assess cell migration, 1.0 × 10^5^ A431 cells transfected with siNC or siBAX_01 or siBAX_02 were seeded into the 8-μm upper chambers of 24-well plates for transwell assay (Cat. No. MCEP24H48, Millipore) in serum-free DMEM. During culture at 37 °C for 18 h, the cells in the upper chambers were attracted by the culture media in the lower chamber, through chemoattractants provided by the included 10% FBS. The chambers were washed with PBS twice and fixed with 3.7% formaldehyde. Cells were permeabilized using 100% methanol at RT, stained with 0.1% crystal violet, and observed using a microscope after being scraped off with cotton swabs.

### 4.11. Cell Invasiveness Assay

For the assessment of invasive ability, Matrigel-coated chambers (Cat. No. 354480, BD Biosciences, San Jose, CA, USA) were used to culture transfected A431 cells. 1.0 × 10^5^ A431 cells transfected with siNC or siBAX_01 or siBAX_02 were seeded into the 8-μm upper chambers of 24-well plates during culture at 37 °C for 24 h. Other treatments were performed as described in the kit manual.

### 4.12. Xenograft Tumor Model

RNA interference (RNAi) oligonucleotides designed to target *BAX* were synthesized by RiboBio Co. (Guangzhou, China), with the sequence 5′-gcucugagcagaucaugaa-3′. Briefly, 1 × 10^7^ cells were subcutaneously implanted into the left and right flanks of female athymic nude mice (4–5 weeks old). At eight days after implantation, siNC or siBAX oligos was injected into the left or right tumor, respectively, and the injection was repeated every other day. Oligos were prepared by pre-incubating 3 nM siRNA per mouse with Lipofectamine 2000 (Life Technologies) for 15 min; injections were made using a final volume of 50 µL in serum free DMEM (Life Technologies). The tumor diameters were measured and recorded every other day to generate a tumor growth curve. After tumor growth assessment, the tumors were excised and snap-frozen for RNA and protein extraction, or paraffin-embedded for IHC staining.

### 4.13. Statistics Analysis

Statistical tests were performed for independent-samples with an unpaired *t*-test, one-way ANOVA tests, or repeated measures tests (SPSS version 19.0, SPSS Inc., Chicago, IL, USA). All statistical tests incorporated two-tailed tests and homogeneity of variance tests, and were considered to reflect significant differences if *p < 0.05, ** *p* < 0.01, or *** *p* < 0.001.

## 5. Conclusions

In summary, our study clearly confirms an association between miR-365 and its downstream target, BAX by in vitro and in vivo expression. The downregulated expression of BAX is significantly associated with the malignant phenotype of CSCC and might serve as a predictor of CSCC together with aberrantly-expressed miR-365. Subsequent mechanistic studies, based on the loss of BAX by RNA interference, and the gain or loss of miR-365, have proved the functional connection dominated by the miR-365-BAX axis in the cellular phenotype (including proliferation, migration, and invasiveness capabilities), which may provide potential targets for developing novel anti-cancer drugs and contribute to establishing new therapeutic regimes.

## Abbreviations

CSCC	Cutaneous squamous cell carcinoma
BCL-2	B cell leukemia/lymphoma 2
BAX	BCL2 associated X, apoptosis regulator
UV	Ultraviolet
IHC	Immunohistochemistry
